# A predictive nature for tactile awareness? Insights from damaged and intact central-nervous-system functioning

**DOI:** 10.3389/fnhum.2015.00287

**Published:** 2015-05-19

**Authors:** Lorenzo Pia, Francesca Garbarini, Dalila Burin, Carlotta Fossataro, Anna Berti

**Affiliations:** ^1^SAMBA (SpAtial, Motor and Bodily Awareness) Research Group, Psychology Department, University of TurinTurin, Italy; ^2^Neuroscience Institute of Turin (NIT), University of TurinTurin, Italy

**Keywords:** sensory expectancies, predictions, tactile awareness, proactive brain, Bayesian brain

## Abstract

In the present paper, we will attempt to gain hints regarding the nature of tactile awareness in humans. At first, we will review some recent literature showing that an actual tactile experience can emerge in absence of any tactile stimulus (e.g., tactile hallucinations, tactile illusions). According to the current model of tactile awareness, we will subsequently argue that such (false) tactile perceptions are subserved by the same anatomo-functional mechanisms known to underpin actual perception. On these bases, we will discuss the hypothesis that tactile awareness is strongly linked to expected rather than actual stimuli. Indeed, this hypothesis is in line with the notion that the human brain has a strong predictive, rather than reactive, nature.

## Introduction

The notion of predictive brain is progressively coming to the forefront of cognitive neuroscience. According to it, the human brain should be conceived not only as a passive receiver of sensory information but also as an active predictor of incoming signals. The brain would continuously generate internal representations of future states in terms of short-term estimations of upcoming events, or long-term guesses about the likelihood of events in the very far future. Then, these predictions wouldbe constantly compared with actual perceived states. Such process would also allow a continuous update of prior knowledge and would let possible to learn from previous experiences (Friston, 2005; Friston et al., 2006). Accordingly, terms as foresight, expectation or anticipation are largely employed to pinpoint predictive processes within distinctive cognitive domains: motor control (Wolpert et al., 1995), self-recognition (Apps and Tsakiris, 2014), motor awareness (Blakemore and Frith, 2003), body ownership (Ferri et al., 2013), social interactions (Brown and Brüne, 2012), perception (Friston and Kiebel, 2009) and learning (Schultz and Dickinson, 2000). Additionally, it has been suggested that the malfunctioning of these predictive processes may partly explain the symptomatology of a number of psychiatric and/or neurological disorders: autism (Pellicano and Burr, 2012; Lawson et al., 2014), schizophrenia (Picard and Friston, 2014) and unawareness of motor (Garbarini and Pia, 2013; Fotopoulou, 2015) or tactile (Pia et al., 2014a,b) deficits.

In the present paper, we will focus on the somatosensory domain, specifically on touch. We will briefly review scientific evidence coming from both intact and damaged central-nervous-system suggesting that predictive processes have a key role in the subjective experience of touch.

## Touch as a Pure Mental Content

The experience of touch is subjectively felt as coherent and self-evident because there is often a strict correspondence between the actual presence (or absence) of a stimulus, and the actual presence (or absence) of its subjective counterpart. However, sometimes we can have a tactile experience in absence of any physical stimulation. Most of us, for instance, have experienced the (false) belief of touch during the transition from wakefulness to sleep or *vice-versa*. Most importantly, recent behavioral, physiological, and neuropsychological findings have reported several conditions in which the sensation of touch is clearly only in the mind of the beholder.

One of the most common situation in which touch does not correspond to physical stimulation is the false tactile perception (i.e., tactile hallucination), in which people may experience touch sensation in absence of any kind of external stimulus (for a review see Berrios, 1982). Hallucinations have been reported in different neurological and psychiatric disorders: psychotic states (Lewandowski et al., 2009), Parkinson’s disease (Fénelon et al., 2002), dementia (Fénelon and Mahieux, 2004), phantom limb (Ramachandran and Hirstein, 1998) and drug abuse (Morani et al., 2013). However, the most convincing evidence about the predictive nature of touch comes from the illusory experience of tactile perception generated by an external stimulus delivered in a different sensory modality. It is worth noticing that tactile illusions differ in nature from those conditions in which tactile perception, rather than being illusory, is boosted by stimuli presented in a different sensory modality (i.e., visual enhancement of touch; see Halligan et al., 1997; Rorden et al., 1999; Taylor-Clarke et al., 2002; Longo et al., 2008).

The vast majority of tactile illusions have been reported with stimuli presented in the visual domain. An actual experience of touch entirely triggered by a visual stimulus can be a relatively common phenomenon even in healthy population. For instance, it has been shown that under certain circumstances people can produce false tactile alarms following a purely visual stimulation (Lloyd et al., 2011; McKenzie et al., 2012). Such a visual dominance over touch is more evident in those pathological conditions in which tactile perception is prevented because a physical damage has directly affected tactile processing. For instance, some brain-damaged patients with a complete loss of contralesional tactile perception (hemianesthesia) may report of being still able to perceive touch, showing what is known as anosognosia for hemianesthesia (Vallar et al., 2003; Marcel et al., 2004; Bottini et al., 2009; Pia et al., 2014a,b). Crucially, they may also report a tactile sensation when they see a tactile stimulus delivered to their anesthetic body parts, where tactile processes cannot occur (Pia et al., 2014a,b). Such a response seems to reflect a real tactile experience rather than a mere verbal confabulation and/or an “as if” situation. Indeed, these patients show a normal anticipatory skin conductance response to the incoming tactile stimuli (Romano et al., 2014). Another interesting instance of tactile illusions can be observed in synesthesia, that is a pathological condition in which a stimulation in one sensory modality automatically induces a conscious sensory experience in a different modality (Watson et al., 2014). Crucially, it has been reported the existence of a mirror-touch form of synesthesia: there are people who experience a tactile stimulation on a given part of their body when they see another individual being touched on the same body part (Blakemore et al., 2005; Banissy et al., 2009; Holle et al., 2011).

Given that tactile perception is body-related, it has been investigated whether and how visual representations of one’s own body play a role in the emergence of tactile illusions. A number of studies have demonstrated that around 30% of normal subjects report tactile sensations on their own hand when a fake (rubber) hand is located very close/superimposed to one’s own hidden hand (Durgin et al., 2007). Additional evidence comes from experimental manipulations in which the physical constraints subserving body representation are manipulated. To this respect, the most solid experimental paradigm is known as the rubber hand illusion (Botvinick and Cohen, 1998; Ehrsson et al., 2004; Tsakiris and Haggard, 2005). In brief, such paradigm shows that synchronous touches onto a visible rubber hand and onto the hidden participants’ hand induce a vivid feeling of ownership of the fake hand both subjectively (as assessed by a self-report questionnaire) and objectively (the location of one’s own hand is reported as being shifted towards the rubber hand). Recently, a modified version of this paradigm employed approaching visual stimuli, that is each stimulus did not touch the rubber hand (Ferri et al., 2013). Results show that people still experience the illusion, exactly as in the classical version in which stimuli are actually delivered to the rubber hand. The subjective report of the illusion can be considered as a veridical experience of touch since it was accompanied with compatible skin conductance responses to the incoming tactile (seen) stimulus. The rubber hand illusion paradigm has been employed also in patients with traumatic spinal cord injury, a pathological condition in which processing of tactile information can be lost. Tidoni and coworkers (Tidoni et al., 2014), for instance, reported a patient who was still capable to experience the rubber hand illusion for the deafferented body parts due to a massive effect of vision. Such a strong influence of vision upon touch is confirmed by the fact that tactile awareness in a complete insentient finger contiguous to a sentient finger can be improved by the rubber hand illusion (Lenggenhager et al., 2012, 2013). Interestingly, this datum is suggestive of the presence of a remapping effect, induced by the illusion, which could trigger a normal body representation of an otherwise numb body part. A sort of permanent rubber hand illusion can also be seen in a number of patients with a particular neurological syndrome, that is patients who mistake someone else’s arm for their own despite unambiguous evidence of the contrary (embodiment phenomenon; for details see (Garbarini and Pia, 2013; Garbarini et al., 2013, 2014, 2015; Pia et al., 2013a). Crucially, and in line with the content of the delusion, tactile stimuli delivered to the embodied body part are subjectively perceived as delivered on the own body (Pia et al., 2013a; Garbarini et al., 2014). Additionally, at physiological level the verbal report is accompanied by arousal responses similar to those registered for the own hand in healthy subjects (Garbarini et al., 2014). Since the prerequisite for the emergence of such tactile illusion is that the stimulus must be seen, this monothematic delusion of body ownership represents another instance of tactile experience induced by visual stimulation. Finally, another kind of tactile illusion has been demonstrated in phantom limb patients. These patients may report pain modulation when they see their unaffected hand superimposed on the amputated one in a mirror (Ramachandran and Rogers-Ramachandran, 1996; MacLachlan et al., 2003) or when they can control a virtual limb in immersive virtual reality (Murray et al., 2007; Cole et al., 2009; Sato et al., 2010).

## A Predictive Account of Tactile Awareness

The aforementioned evidence shows that veridical tactile sensations can arise in the absence of any tactile stimulation. In order to explain this phenomenon, we need to understand which are the neurofunctional mechanisms subserving tactile awareness.

It is known that tactile information reaches the primary somatosensory cortex through the thalamic nuclei. BA3b, a sub region of the primary somatosensory cortex, represents the first stage of tactile processing and it is able to detect stimulus intensity and even nociception. The second stage occurs in BA1. Subsequently, both BA3b and BA1 transmit information to BA2, the third stage of tactile processing in which visual, auditory and somatosensory signals are integrated. From the primary somatosensory cortex, the information is sent through reciprocal connections to the secondary somatosensory cortex, the fourth level stage of information processing. The current neurocognitive model of tactile awareness in humans (e.g., Gallace and Spence, 2008) states that when the reciprocal integration between primary/secondary somatosensory cortices and higher order structures is achieved, conscious awareness can start to arise. It is worth noticing that only higher processing stage activities (e.g., BA2, SII) are present when touch is observed on another person (Keysers et al., 2004). Hence, it is possible that earlier stages of somatosensory operations (e.g., BA3b) are involved only in the processing of signals coming from one’s own body, whereas later processing stages alone could be activated when we observe other people being touched (Schaefer et al., 2009). At this point, signals are progressively integrated with the involvement of several higher order areas as parietal cortex, insula and even motor areas.

The aforementioned model of tactile awareness (Gallace and Spence, 2008), allows a clear-cut prediction: tactile awareness can in principle arise even in absence of any tactile stimulus delivered to one’s own body. The literature reviewed above is consistent with this notion. Indeed, tactile hallucinations (Huber et al., 1984; Shergill et al., 2003), false alarms in healthy subjects (Lloyd et al., 2011), false beliefs of perceiving actual tactile stimuli in anosognosia for hemianesthesia (Pia et al., 2014b) and synesthetic touch (Blakemore et al., 2005) are, in a way or another, linked to activities of at least earlier somatosensory cortices (but even higher order areas), but in absence of any physical (tactile) counterpart.

A further issue concerns the nature of the processes leading to tactile awareness. It is known that the human brain has a multisensory signature. In other words, when an incoming input has a high certainty in one given sensory modality, it can modulate perceptual consequences in a different modality (Driver and Spence, 2000). However, the nature of tactile illusions requires a mechanism responsible for triggering tactile awareness in absence of tactile stimulation, but in presence of a stimulus in another modality (suggestive of a possible incoming tactile simulation). According to the notion of the predictive brain mentioned above Bar (2007), such a mechanism could be conceived in terms of a predictive model. Since biological systems must face the uncertainty of the environment in which they live, the most adaptive responses are those who succeed in minimizing the cost of the surprise effect. The best way to achieve this is developing a system capable to anticipate the most probable events in a certain context. This, in turn, would allow the selection of the optimal behaviors, namely those which improve the subsequent stimulus detection (e.g., Brunia and van Boxtel, 2001; Bausenhart et al., 2006) and its processing (Desimone and Duncan, 1995). With respect to touch, former studies on the anticipation of tactile stimuli reported activations in somatosensory cortices, in the same areas that subserve tactile perception (Drevets et al., 1995; Carlsson et al., 2000). More interestingly, a recent fMRI study (Langner et al., 2011) has tried to isolate the activation related to pure expectancy. The authors analyzed the functional activations only in those trials in which the cue indicating the modality of the upcoming target was not followed by any stimulus. Results show that explicit tactile expectations are underpinned by an anticipatory increase of the baseline activity within relevant primary/higher order sensory cortices (i.e., primary and secondary somatosensory cortices), and a decrease within irrelevant primary/higher order sensory cortices (primary and secondary visual and auditory cortices). For our purpose, the key finding is that the human brain can represent in advance tactile-specific information not only within tactile-specific areas but also in non-tactile areas. This finding suggests that early sensory cortices, traditionally regarded as unisensory, can also process multisensory information. It is worth noticing that this latter idea is in line with the notion that stimulation in a given modality also induces activities in early sensory cortices specific for other modalities (Meyer et al., 2011; Vetter et al., 2014; Smith and Goodale, 2015).

**Figure 1:**
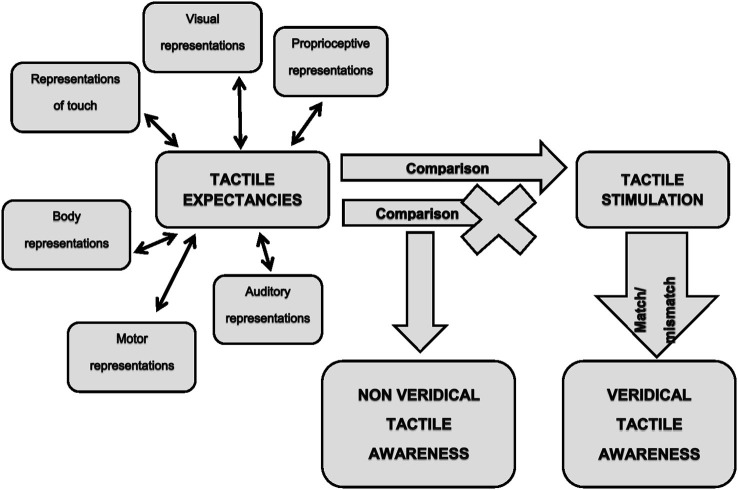
**Represents a sketch of the proposed model of tactile awareness in humans**.

On these bases, we believe that the previously mentioned neurocognitive model of tactile awareness (Gallace and Spence, 2008) could be integrated with the concept of tactile expectancies to explain most of the visually-triggered tactile illusions. This in turn, would allow gaining further insight into the mechanisms underpinning tactile awareness in humans. As noted above, the brain continuously transforms relevant stimuli in tactile expectancies in order to produce the best response under certain circumstances. Such expectancies would be generated within the complex interplay between low and higher order sensory cortices and would represent the neural signals on which tactile awareness would emerge (Figure 1, left part). It is worth noticing that expectancies could be generated not only on the basis of visual stimulation, but also on the basis of any sensory information relevant for the emergence of sensory awareness. For instance, it is known that mirror-touch synesthesia (Banissy et al., 2009; Holle et al., 2011), delusional body ownership (Pia et al., 2013a) and tactile illusions in spinal cord injury (Tidoni et al., 2014) occur when observing touch delivered on the human body but not on objects or dummies. This means that stored internal representations impose constraints on the emergence of a specific tactile experience. Since the human body receives tactile stimuli also during actions, information arising from the motor system should be included among the source of signals for expectancy generation. The fact that in the normal wake state it is nearly impossible to tickle oneself (Blakemore et al., 1998), demonstrates that internal representations of willed actions do affect tactile processing. Expectancies would then be compared with the actual sensory feedbacks (Figure 1, middle part). The detection of the match (stimuli are present)/mismatch (stimuli are absent) between expected and delivered stimuli should lead to a veridical tactile awareness (Figure 1, right lower part). We suggest that visually triggered tactile illusions arise from a defective detection of the mismatch between expected and actual stimulations (Figure 1, middle part). Hence, in this case tactile awareness would entirely relies on expectancies, with the consequence of experiencing illusory touch in absence of an actual tactile stimulation (Figure 1, middle lower part).

It is worth noticing that our model for tactile awareness is similar to the one we proposed to explain the experience of illusory movements in patients with anosognosia for hemiplegia (Berti and Pia, 2006). In that case, the false belief of being still able to move is seen as a failure to detect the mismatch between intended and actually executed movements (e.g., Jenkinson and Fotopoulou, 2010; Pia et al., 2013b; Piedimonte et al., 2015). Additionally, the hypothesized role of predictive mechanisms in building conscious awareness could be extended to sensory domains other than touch. As regards vision, for instance, there is evidence of functional interactions between visual and prefrontal areas (Kveraga et al., 2007; Axmacher et al., 2008) and prediction-based prefrontal modulations of early visual processing has been described (Kveraga et al., 2007; Gamond et al., 2011). Accordingly, it has been suggested that such reciprocal connections might subserve the comparison between actual and predicted visual stimuli. This predictive mechanism would quickly enable the content of visual awareness, thus reducing uncertainty (Panichello et al., 2013). This hypothesis is line with evidence showing that predictions do affect the perceptual content. It is known, for instance, that a visual prime related to one interpretation of an ambiguous figure significantly biases perception towards that interpretation (Goolkasian and Woodberry, 2010). Similarly, successful perception of fragmented object figures is more likely when the observer is informed about the semantic category of the object (Reynolds, 1985). Moreover, when visual stimuli with altered/ambiguous edges move smoothly in space, we tend to report their location in advanced positions (Soga et al., 2009).

In conclusion, although we believe that the reported evidence supports the notion of the predictive nature of tactile awareness, further behavioral, physiological and anatomo-functional evidence is still required. Indeed, this interpretation is primarily based on the explanation proposed for tactile illusions reported in anosognosia for hemianesthesia. These patients, despite never referring of being touched on the affected side during the standard neurological examination with their eyes closed, they report touch when they see a stimulus delivered to their anesthetic body part. This disturbance is explicitly interpreted as a neurologically-based failure to detect the mismatch between visually-triggered tactile expectancies and the actual absence of tactile stimulation (Pia et al., 2014a,b). However, the role of residual bottom-up tactile processing in the emergence of such illusory experience of touch should be clearly excluded. In other words, direct electrophysiological measures (e.g., somatosensory evoked potentials), for instance, should confirm the complete absence of touch-related electrophysiological activity from the periphery of the somatosensory system. If this is the case, a clear prediction can be put forward: the illusory perception of touch should be subserved only by top-down activities triggered by the visual modality in spared primary and/or secondary somatosensory cortices Finally, a further issue concerns the investigation of the evolutionary significance of predictive mechanisms. In other words, it would be crucial to study the role of such predictive mechanisms in an ontogenetic and/or phylogenetic perspective.

## Conflict of Interest Statement

The authors declare that the research was conducted in the absence of any commercial or financial relationships that could be construed as a potential conflict of interest.
